# Analysis of the three-year work of a digital pathomorphological laboratory built from the ground

**DOI:** 10.1016/j.jpi.2022.100111

**Published:** 2022-07-05

**Authors:** Rudenko Ekaterina Evgenievna, Demura Tatiana Alexandrovna, Vekhova Ksenia Andreevna, Lobanova Olga Andreevna, Yumasheva Valentina Alekseevna, Zhakota Dmitrii Anatolevich, Anoshkin Kirill, Remez Alexey, Untesco Maksim, Kroman Nikolay, Mayer Artem, Zhuravlev Alexander, Kryatova Alexandra, Lyapichev Kirill, Genis Mikhail

**Affiliations:** a Sechenov First Moscow State Medical University (Sechenov University), Trubetskaya street, 8, p. 2, 119991 Moscow, Russia; b Department of Pathology, Pediatric Faculty, Pirogov Russian National Research Medical University (Pirogov Medical University), Moscow, Russia; c Research Centre for Medical Genetics, Moskvorechye str. 1, 115522 Moscow, Russia; d Unim LLC, Podsosenskaya lane, 23, b.6, 105062 Moscow, Russia; e The University of Texas Medical Branch, 301 University Blvd, Galveston, TX 77555, USA

**Keywords:** Telepathology, Digitization, Pathomorphology, Digital, Pathology, Cancer, Oncology, Automation, Optimization, MDT, Consultation, Telemedicine, Digitalization, Diagnosis, Cost-effectiveness

## Abstract

Digital pathology is a new stage in the development of pathomorphological diagnostics. This topic was most widespread during the COVID-19 pandemic. The advantages of digitization of diagnostics include the possibility of remote work of a pathologist, remote asynchronous consultation, and automation of business processes. They provide an increase in diagnostic quality and speed up the diagnosis process. These benefits are only a small part of what digital cancer diagnostics can provide. This article is written on our own experience of Russia's first fully digital pathomorphological laboratory UNIM. All advantages and disadvantages of digitization, peculiarities of using technology, differences from the conventional approach to diagnostics, the economics of the process, the importance of integration with LIS (laboratory information system) and MIS (medical information system), errors and principles of their solution, payback will be discussed, and every stage of laboratory work will be considered in detail: from logistics and registration to diagnosis and archiving. Due to the fact that all data has been digitized over several years, we will present a comprehensive analysis of statistics and observations on how to organize processes in a fully digital laboratory. A key feature of our experience is the high cost-effectiveness of the platform and approach, which allowed us to win the competition in the market. The result of the survey of doctors' attitudes towards digital pathology will also be presented.

## Introduction

The use of digital methods in pathomorphology expands not only the pathologist’s tools but also the capabilities of all the processes involved in making a diagnosis. Partial or sometimes complete automation of these processes increases productivity, quality, and controllability of the diagnosis process. The possibility of an "easy" consultation with the involvement of a "second opinion" in each case reduces the likelihood of error. The remote availability of pathologists of the relevant subspecialty is a guarantee of improving the quality of the entire process. Of great importance is the accumulation of data, opportunities for learning and contextual search, as well as image-based search.

Currently, there are not many materials devoted to the comprehensive analysis of the work of fully digital pathomorphological laboratories and the accumulated statistical data on their work and analysis of the economics of the pathomorphological laboratory particularly.^20^^,^^21^ Certain articles describe the economic efficiency of introducing digitization into the diagnostic process.^22^ Several studies are aimed at calculating cost reduction and improving work efficiency.^23^^,^^24^^,^^25^ None of the presented studies includes the whole range of issues of interest: a complete description of all the processes and stages of the digital pathomorphological laboratory, work experience, disadvantages and advantages of technology implementation, economic calculations, and the opinions of specialists working in digital. The lack of information is still determined by the lack of practical experience and the low number of laboratories working fully digitally.

## Materials and methods

In early 2018, Russia's first fully digital pathomorphological laboratory, UNIM, was launched at the Skolkovo Innovation Center Technopark in Moscow, where the entire material stream was digitized. The project was commercial, launched on the basis of laboratory information system (LIS) of our own design, covering all stages in pathomorphological diagnostics: preanalytical phase (customer portal and laboratory information system), analytical as well as post-analytical phases (digital pathology (DP) platform). The LIS also includes a set of statistical tools (mission control center (MCC) and partially DP platform) and communication tools (Slack, corporate Gmail). All components of the LIS are fully integrated with Digital Pathology with the ability to transmit information in real-time and in both directions (bidirectional).

The LIS system provides different notification methods for all participants in the process:

(a.) case ready for review when additional information becomes available;
(b.) in the case of a request from other participants in the process (for example, a request to the clinician is addressed from the chat room attached to each case, via messengers automatically);
(c.) if necessary to take action based on timers and reminders.

The company is currently working on the implementation of an automatic pathologists assignment procedure.

After the validation of the diagnosis in case a team of specialists worked on it or after signing the diagnosis by one pathologist if the case belongs to a routine one (see the rules for appointing a Concilium see the section on appointing a pathologist), the report is automatically sent.

All doctors use an enhanced qualified electronic signature, which is legally equivalent to a handwritten signature.

The study analyzed 3 years of work (2018–2020) of the digital laboratory: 107 270 cases, 222 923 created and 52 876 incoming blocks, 310 868 created and 63 122 incoming slides, 126 006 raw material and 366 842 scanning operations.

## Results

### Logistics and cases

At the moment of the beginning of 2021, the laboratory serves the whole territory of Russia. The logistical complexity of working with the Moscow and Moscow region is less than in other regions: all material from Moscow and Moscow region (radius ~ 110 km) is delivered by our own courier service. The average cost of delivery per case in Russia at the end of 2020 is 1,3$. And in 3 years, it has increased by 6%.

For this period, we worked with 227 clinics: 96 private and 131 governmental ones.

The average amount of cases from 1 clinique is estimated as 350/year.

The cases include biopsy material, surgical material.

10% cases require immunohistochemical analyses with from 1 to 43 different antibodies (average is 5,6 antibodies per case)

Most of the cases from the regions are complex cases, while the entire flow, including the primary flow, comes to the company from the Moscow region (Table 1). Over time, the company spreads its influence and strengthens its reputation, which is reflected in the growth of complex cases from the regions (more and more state medical institutions apply to the company for the opportunity to provide an expert opinion with the participation of relevant specialists).

**Table 1:** Cases distribution by regions and complexity over recent years.

Regional distribution of cases	2018	2019	2020	Overall
	%(n)	%(n)	%(n)	%(n)
Moscow and Moscow region	98% (9518)	96% (39393)	89% (49969)	92% (98880)
Complicated cases^a^	28% (2685)	19% (7335)	19% (9621)	20% (19641)
Primary cases	72% (6833)	81% (32058)	81% (40348)	80% (79238)

Other regions	2% (221)	4% (1843)	11% (6326)	8% (8390)
Complicated cases	44% (95)	74% (1372)	80% (5049)	78% (6516)
Primary cases	56% (126)	26% (471)	20% (1277)	22% (1874)

a Complicated case is one with revision, oncology, or a case where there has been a consultation.

Most of the regional clients have state ownership, while the share of private companies in the regions is growing (Table 2).

**Table 2:** Statistics by the form of ownership of client clinics by per cent of cases by year.

Distribution of cases by form of ownership of referring clinics	2018	2019	2020	Overall
*Moscow and Moscow region*				
State property	0,0%	2,2%	3,1%	2,4%
Private property	100,0%	97,8%	96,9%	97,6%

*Other regions*				
State property	99,5%	80,7%	71,2%	74,1%
Private property	0,5%	19,3%	28,8%	25,9%

The disproportion between different forms of ownership in Moscow and the regions can be explained by the following factors:

- The market for commercial services in the field of pathomorphological research in Russia is underdeveloped: barely 5%–10% of all studies are conducted on commercial terms, in 90% of cases the state is involved in the treatment of oncology.

- Commercial

Absolutely all indicators of workload per employee (one of the main performance indicators) are growing steadily (Table 3).

**Table 3:** Load dynamics.

Load indicator	2018 n	2019 n (%YoY^a^)	2020 n (%YoY)
Number of cases per year per laboratory employee	755	2 059 *(273%)*	2 447 *(119%)*
Number of cases per year per lab technician	1 091	3 743 *(343%)*	4 690 *(125%)*
Number of cases per year per pathologist	1 403	3 743 *(267%)*	4 329 *(116%)*
Average daily number of scanned slides	136	481 *(354%)*	699 *(145%)*
Average daily number of scanned slides per laboratory employee	10	24 *(240%)*	30 *(125%)*
Maximum number of daily scanned slides per year	560	927 *(166%)*	1 210 *(131%)*
Same per laboratory employee	43	46 *(107%)*	53 *(115%)*

a YoY - Year-Over-Year - a comparison for comparing events on an annualized basis.

This is due to the following factors:

1. The overall absolute increase in the volume of cases allows for more even use of available resources.
2. Continuous optimization of business processes and automation also have a significant impact on resource efficiency.

The diagnosis process was originally created on the basis of the consultation required in every non-routine case or case with any signs of malignancy. In 3 years, 32% (n= 34 362) of cases required the involvement of several specialists. In 85.69% (n = 29 446 ) it took 2 pathologists to make a diagnosis, in 12.36% (n = 4246 ) - 3 and in 1.95% (n = 670) more than 4 specialists.

A consultation involves 2 or more specialist-pathologists. At least one of the participants must have an appropriate specialization.

To improve the quality of diagnosis and obtain patient information quickly, clinical specialists of the referring patient are included in diagnostic chats MDT. This is done either at the initiative of the pathologist performing the diagnosis or at the initiative of the referring clinic: some clinics ask to include their clinical specialists in all cases with their patients.

### Way of assignment of pathologists and having consultations

When a case is ready for diagnosis: slide scans and clinical information are uploaded; a pathologist is assigned by the laboratory administrator, taking into account the sub-specialization, the daily case rate and availability. The administrator appoints 2 pathologists at once if at least one of the conditions is fulfilled: the case is a revision or surgical material in case the clinical diagnosis indicates malignant tumor or any other significant pathology or no pathologists with the appropriate subspecialty is currently available.

The same pathologist who looked at the original case is assigned to the patient's previous cases (assuming the pathologist's narrow profile and access).

When 2 pathologists are assigned, one of them is assigned the role of writing the report, the other pathologist validates.

If the medical specialists do not come to a consensus as a result of discussing the case in the case’s chat, they ask for a third pathologist to be involved. The maximum number of medical practitioners involved in the diagnosis in our experience was 15.

If a consensus is reached, recorded in the chat, 1 pathologist writes the report using appropriate templates, and the other pathologists who participated in the diagnosis validate it, indicating their agreement with the diagnosis.

An extremely important fact is that the commercial component in assigning an additional pathologist to a case does not influence this decision. Pathologists do not have a key performance indicator (KPI) for the cost of a case, but they do have a KPI for errors.

### Employment of pathologists

Statistics of test case responses:

- Number of candidates who answered the cases: 100;

- Number of cases per candidate: 3 (before 2019) to 5 (after 2019);

- Assessment of correctness of the answer, in points: from 1 to 5;

- Number of accepted specialists: 65. At the beginning of 2021, there were 12 full-time pathologists, and others were for partial work.

The average number of cases per pathologist is (cases/day):

-Full-time employment: 20–30 cases/day;

-Part-time employment: 5–15 cases/day;

The average number of working days: 5.

Duration of the consultation: 1–4 days.

### Internal audit

The company conducts internal audits on a daily basis. The procedure is as follows:

1. The administration randomly selects 2% of the cases for which opinions were sent on the previous day.

2. These cases are referred to the auditors for review by the appropriate nosology.

3. The chief pathologist decides who to involve in the review additionally.

4. Those pathologists who participated in the diagnosis cannot be involved in the auditing.

5. As a result of the review, the 2 pathologists should have the same opinion about the revised case.

The following options are possible:

a. Two opinions coincided with the indicated diagnosis. The case passed verification.

b. In all other cases of any discrepancy (either the diagnosis of 1 of the 2 reviewing pathologists, or both with the original diagnosis) - the pathologist who validated the report is involved in the discussion, then the discussion results in a final, agreed by all participants of the diagnosis process: whether there was an error, the degree of its criticality, the reasons for it (about the reasons for errors see "Quality control of diagnostics").

The degree of criticality of an error for statistical purposes is divided into

1. Critical - affects therapy/prognosis

2. Non-critical - does not affect therapy/prognosis

Based on an analysis of the causes of the error, if appropriate, conclusions are drawn about the causes and possibilities of eliminating these causes by organizational methods or using digital technology are considered. For example, a pathologist made a mistake by omitting several tumor cells in a specimen (a few cells out of 10 000). This led to the decision to create a dedicated tool that checked the pathologist to determine whether he/she had missed any areas that the instrument deemed suspicious. In the future, if enough accuracy is achieved in determining "suspicious" cells it is planned to demonstrate such areas to the pathologist in advance. For now, the tool is still being finalized and will be tested in various modes.

### Cost analysis

Operational (current) payback of the laboratory was achieved a year and a half after opening at 700 cases per month. The payback in this case is calculated as the difference between all revenues and all expenses directly related to the diagnostic process. Administrative, Research and Development (R&D) costs were not taken into account. The company reached full profitability, including all costs, including R&D, in 2 years from the launch of the first laboratory. The operating margin, depending on the case, ranges from 15% for the most inexpensive studies (gastric biopsy specimens) to 50% for complex cases. The average operating margin is about 40%.

**Fig. 1:**
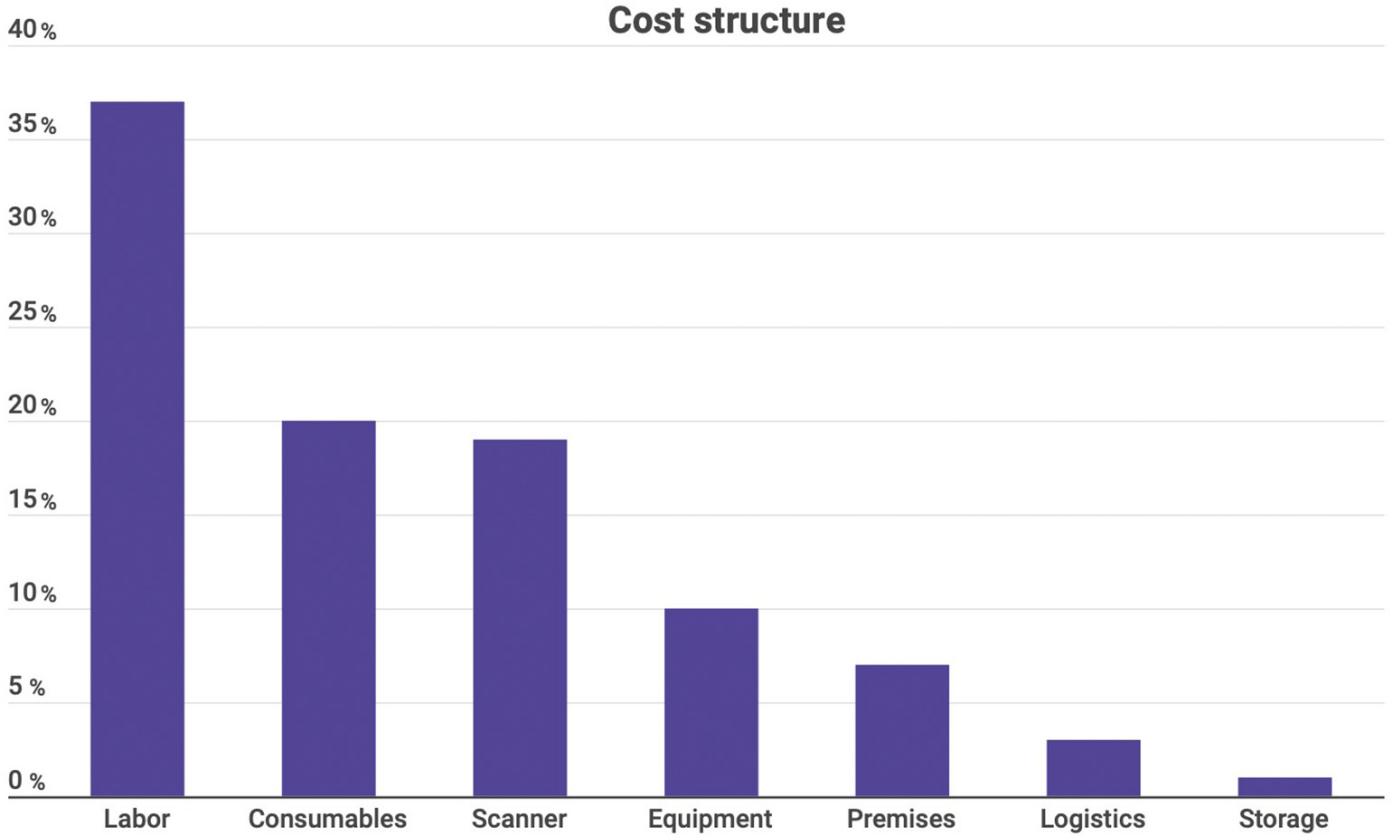
Share of types of expenses in the average case.

The share of each of the presented types of expenses in the average case is presented in Fig. 1.

As can be seen from the presented diagram, labor makes up the largest share of all costs in an average pathomorphological study (which, of course, is not surprising, even taking into account the partial automation of working time and full automation of the document flow).

**Fig. 2:**
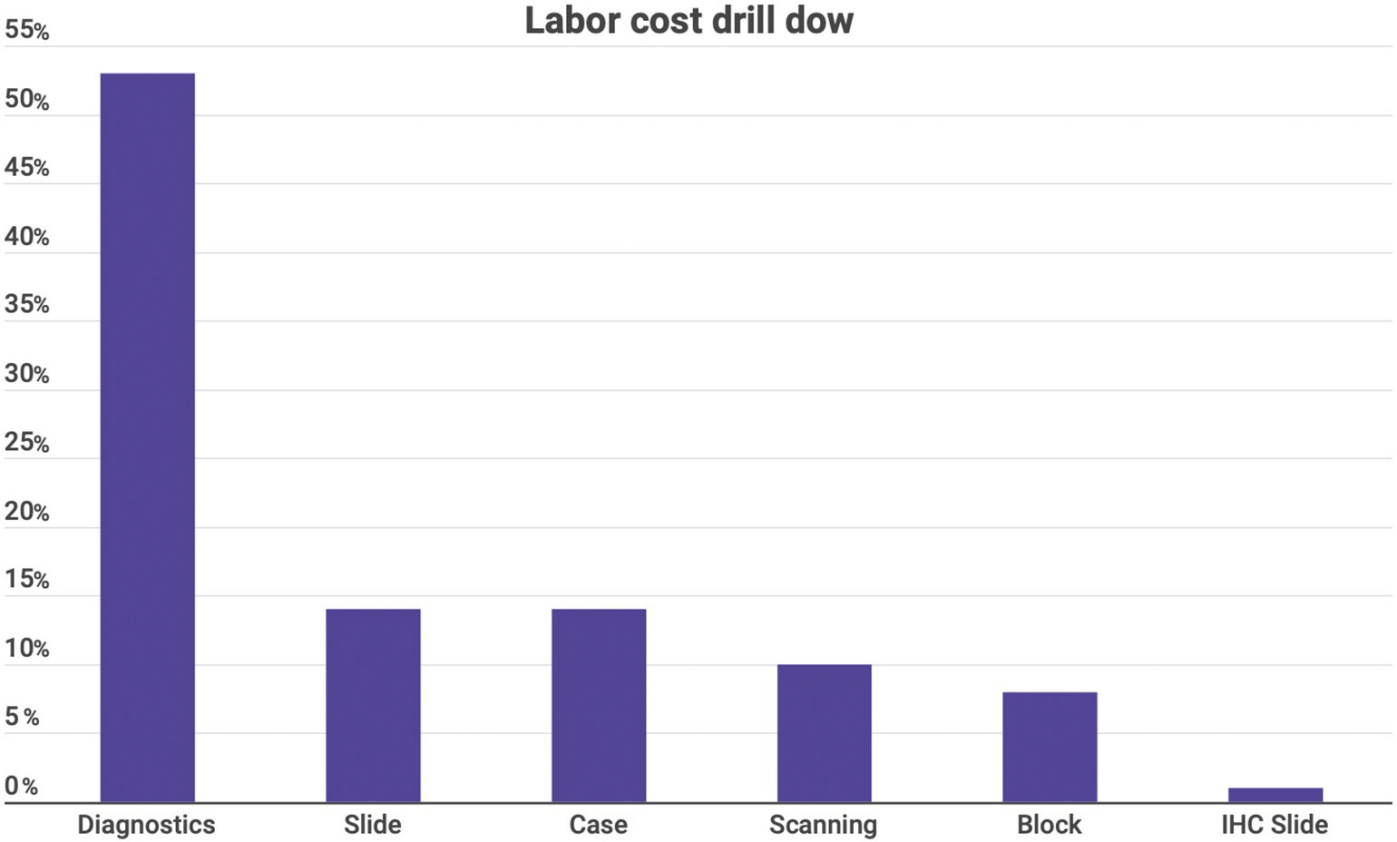
Percentage of labor costs for each stage in the average case.

The percentage of labor costs for each of the stages is presented in Fig. 2.

## Discussion

Increasing the productivity of 1 employee and 1 piece of equipment is necessary to increase the economic efficiency of the laboratory as a business unit and consequently, this will contribute to the spread of digital technologies more widely.

As in other countries in 2020, Russia saw a decrease in the number of tests relative to the previous year, when the medical system diverted its resources to the pandemic. The use of digital pathology methods allowed the laboratory to take over volumes from those laboratories that were closed for quarantine. Biological material from there went to the UNIM lab, went through all the stages of digitization and necessary research, and the digitized cases were accessed remotely by pathologists at the quarantined labs. This made it possible to continue diagnostics for many laboratories, as well as, to support the flow that was sent from some laboratories of closed medical institutions to serve patients.

### Pros and cons of digital pathology

Along with the obvious advantages of using digital pathology, there are significant limitations that one who is going to implement new methods will have to face.

These restrictions apply to the following areas:

- Financial. Implementation will require partial or complete re-equipment of the laboratory; strengthening of communication channels; costs for reorganization, training, re-configuration, and data storage.

- Processes. Changing familiar processes will require reengineering: modifying existing processes and creating new ones.

- Psychological. Changing such a sensitive field as morphological diagnosis requires determination, persuasiveness, and a willingness to implement fundamentally new methods of work.

- Political. It takes determination and strong motivation to overcome resistance, often not always rational. In addition, increasing the transparency of the process to the level of "absolutely transparent" will also, in some cases, require additional effort.

### Implementation risks

- Lack of financial resources. Initial investments are not small and, as numerous experience shows, it is not necessary that if one buys hardware and software it guarantees the success of the project.

- Indecision. It is easier to do as others do, but so far there is little such experience in the digitalization of pathology, therefore it is not easy to decide.

- Lack of a project team. For the business transformation of existing processes, as well as for the creation, testing, and implementation of new ones, a qualified motivated team is needed. And the most important resource is the project owner/sponsor.

- Insufficient qualifications of the project manager. It is difficult to overestimate the importance of motivation and qualifications of a project manager in such projects.

- Lack of risk control while changing current business processes. Pathomorphological diagnostics is the most important process in the fate of the patient and, consequently, in the work of any medical institution. That is why any changes should be controlled and predictable. That it is worth using the risk management tools approach.

### Vulnerabilities and challenges of digital pathology

Here are some points that should be kept in mind.

- Increased quality requirements for microtomes.

- Additional steps (scanning, storage, and image quality control) reduce overall system reliability.

- Industrial scanning microscopes are quite expensive.

- Big data storage requires non-trivial solutions to optimize access speed and storage costs.

### Risks of using and dependence on the digital technology (reliability of the digital process)

At the moment, pathomorphology is dominated by human factor risk. Digital pathomorphology reduces its importance but instead, there are several additional steps containing risks:

- Risks of scanning equipment failure. Minimized by duplication/reservation/appropriate support contracts for related equipment.

- Risks associated with communication channels. These are minimized by duplicating the channels. It is important to be able to ensure that both communication links (primary and backup) from the scanning location to the storage site do not have common physical points.

- Risks associated with storing digital data in the cloud. A set of measures aimed at complying with and constantly updating information security standards. These are minimized by a professional independent audit and strict compliance with its recommendations.

### Prediction of digitization spread in pathomorphology

We expect significant breakthroughs in the next 5 years and an almost universal transition to digital pathology in 7–10 years.

The vanguard of change will be countries in Asia and Africa, where on the one hand, the problems are much more serious, but on the other hand, the regulatory constraints are not as severe.

Also, more rapid changes can be expected from those countries/regions where the leverage of centralized medicine management is higher.

## Conclusions

Despite the many difficulties associated with the introduction of digitization in pathomorphological diagnosis, this process is irreversible: the convenience and the potential of digitization are not just a basis for believing in the future, but already a basis for concluding in the present. Studying the experience of digitization of laboratories that have gone digital in a variety of ways, involving physicians, managers, economists, and engineers, will provide great room for improvement. The very realization of the opportunity to use the full power of digitalization to improve not only quality diagnostics but also to reduce costs practically proves this statement.

The transition to digital diagnostics is painful and there should be a substantial justification for it.

In this sense, UNIM was lucky to be a Startup that had no other way out in the process of its development, except to prove to the market that digital work is not just obviously better, but competitive concerning the traditional diagnostic method, including economically.
