# Erratum to “Analysis of the three-year work of a digital pathomorphological laboratory built from the ground” [Journal of Pathology Informatics Volume 13, 2022, 100111]

**DOI:** 10.1016/j.jpi.2025.100428

**Published:** 2025-04-26

**Authors:** Rudenko Ekaterina Evgenievna, Demura Tatiana Alexandrovna, Vekhova Ksenia Andreevna, Lobanova Olga Andreevna, Yumasheva Valentina Alekseevna, Zhakota Dmitrii Anatolevich, Anoshkin Kirill, Remez Alexey, Untesco Maksim, Kroman Nikolay, Mayer Artem, Zhuravlev Alexander, Kryatova Alexandra, Lyapichev Kirill, Genis Mikhail

**Affiliations:** a Sechenov First Moscow State Medical University (Sechenov University), Trubetskaya street, 8, p. 2, 119991 Moscow, Russia; b Department of Pathology, Pediatric Faculty, Pirogov Russian National Research Medical University (Pirogov Medical University), Moscow, Russia; c Research Centre for Medical Genetics, Moskvorechye str. 1, 115522 Moscow, Russia; d Unim LLC, Podsosenskaya lane, 23, b.6, 105062 Moscow, Russia; e The University of Texas Medical Branch, 301 University Blvd, Galveston, TX 77555, USA

The publisher regrets that the Declaration of Competing Interests was missing in the published article. The statement should read as follows:

## Declaration of Competing Interest

The authors declare that they have no known competing financial interests or personal relationships that could have appeared to influence the work reported in this paper.

The publisher would like to apologise for any inconvenience caused.

